# Synaptic and cellular changes induced by the schizophrenia susceptibility gene *G72* are rescued by *N*-acetylcysteine treatment

**DOI:** 10.1038/tp.2016.74

**Published:** 2016-05-10

**Authors:** B Pósfai, C Cserép, P Hegedüs, E Szabadits, D M Otte, A Zimmer, M Watanabe, T F Freund, G Nyiri

**Affiliations:** 1 Laboratory of Cerebral Cortex Research, Institute of Experimental Medicine, Hungarian Academy of Sciences, Budapest, Hungary; 2 Medical Faculty, Institute of Molecular Psychiatry, University of Bonn, Bonn, Germany; 3 Department of Anatomy and Embryology, Hokkaido University Graduate School of Medicine, Sapporo, Japan

## Abstract

Genetic studies have linked the primate-specific gene locus *G72* to the development of schizophrenia and bipolar disorder. Transgenic mice carrying the entire gene locus express *G72* mRNA in dentate gyrus (DG) and entorhinal cortex, causing altered electrophysiological properties of their connections. These transgenic mice exhibit behavioral alterations related to psychiatric diseases, including cognitive deficits that can be reversed by treatment with *N*-acetylcysteine, which was also found to be effective in human patients. Here, we show that G72 transgenic mice have larger excitatory synapses with an increased amount of *N*-methyl-d-aspartate (NMDA) receptors in the molecular layer of DG, compared with wild-type littermates. Furthermore, transgenic animals have lower number of dentate granule cells with a parallel, but an even stronger decrease in the number of excitatory synapses in the molecular layer. Importantly, we also show that treatment with *N*-acetylcysteine can effectively normalize all these changes in transgenic animals, resulting in a state similar to wild-type mice. Our results show that *G72* transcripts induce robust alterations in the glutamatergic system at the synaptic level that can be rescued with *N*-acetylcysteine treatment.

## Introduction

Schizophrenia affects ~1% of the adult population.^1^ The disease is characterized by a broad range of positive, negative and cognitive symptoms. The exact pathogenesis of schizophrenia is unknown; however, several signs indicate the role of a disturbed glutamatergic transmission in the prodromal phase of schizophrenia causing memory and concentration deficits, as well as other negative symptoms, which often precede the onset of the disease.^2, 3^ Glutamatergic synapses and *N*-methyl-d-aspartate-type glutamate receptors (NMDARs) are the key participants in these processes,^4, 5^ and NMDAR antagonists reproduce negative, positive and also many of the cognitive symptoms.^6, 7, 8^ Changes in the glutamatergic system can also provoke a hyperdopaminergic state in schizophrenia;^9^ however, amphetamine-induced hyperdopaminergic state causes only positive symptoms.^10^ Because currently used antipsychotics (which antagonize dopaminergic/serotonergic receptors) reduce mainly positive symptoms, the treatment of negative and cognitive deficits is still a big challenge.^11^

Schizophrenia is highly heritable with several susceptibility genes.^12^ One of the most frequently reported associations with the disease is found at a locus on chromosome 13q32-q33 containing two overlapping genes, *G72* and *G30*.^13, 14^ These genes appeared late during primate evolution, and are present only in anthropoid primates.^14^ Analysis of serum and postmortem brain samples of schizophrenic patients revealed an overexpression of *G72*, compared with control subjects.^15, 16^ Function of the longest splice variant, LG72, remains controversial. It has been demonstrated to bind D-amino acid oxidase (DAO), the main degrading enzyme of NMDAR co-agonist D-serine,^17^ resulting in either enhancement or depression of its enzymatic function.^14, 18, 19^ Furthermore, LG72 has been shown to be located in mitochondria, influencing oxidative stress regulation^20^ and affecting mitochondrial morphology and dendritic arborization.^21^

To examine physiological effects of the encoded proteins of *G72*, a ‘humanized' transgenic mouse line was generated, carrying the entire human *G72/G30* genomic region. These transgenic mice (G72Tg) encode splice variants similar to those present in the human brain. G72Tg mice showed behavioral alterations that are expected in a model of schizophrenia.^22^ They had an impaired spatial learning ability, suggesting damaged hippocampal function,^23^ and *G72* mRNA expression was among the highest in the dentate gyrus (DG).^22^ Moreover, electrophysiological data revealed alterations at excitatory perforant path synapses in the DG, where *G72* is expressed both in pre- and postsynaptic cells.^23^ Deformations of DG are reported to be predictive for schizophrenia.^24^ Inadequate maturation of dentate granule cells has been linked to neuropsychiatric disorders,^25^ and further morphological, functional and molecular dysfunctions of DG were observed in patients suffering from schizophrenia^26, 27^ and bipolar disorder.^28^ These disorders share clinical features and genetic vulnerability,^29^ and *G72* polymorphisms also correlate with the onset of bipolar disorder.^30^

Growing evidence indicates the role of oxidative stress in the generation of schizophrenia and bipolar disorder.^31^ In addition, mitochondrial dysfunctions, especially at complex I, are repeatedly observed in these disorders (see Discussion for details). LG72 can bind to complex I, which has a central role in the generation of reactive oxygen species.^32^ Glutathione (GSH) is the main antioxidant of the brain. Compared with healthy control subjects, patients suffering from schizophrenia or bipolar disorder have significantly lower GSH levels.^33, 34^ Interestingly, treatment with *N*-acetylcysteine (NAC), a putative GSH precursor was shown to have beneficial effects on negative symptoms of reported schizophrenic cases,^35, 36^ and on depressive symptoms in bipolar disorder.^37, 38^ Similarly, G72Tg mice have decreased mitochondrial complex I activity and lower GSH levels, and NAC treatment rescued the above-mentioned spatial learning deficit in these G72Tg mice.^23^

In the present study, we reveal cellular and synaptic changes that may explain the spatial learning deficits and electrophysiological alterations of DG in the G72Tg animals. We demonstrate an increase in the size and NMDAR content of excitatory synapses on dentate granule cell spines, which is accompanied by a decrease in the total number of excitatory synapses and granule cells in the DG. Most importantly, oral treatment with NAC almost completely restored these cellular and synaptic changes in G72Tg animals. These results can provide an explanation for the effectiveness of NAC treatment in human cases and can facilitate further research to alleviate negative symptoms in patients suffering from schizophrenia and bipolar disorder.

## Materials and methods

### Animals and tissue preparation

All experiments were performed in accordance with the institutional, governmental and European Union guidelines. G72 transgenic mice (G72Tg) were generated as described before.^22^ The ages of experimental animals were the following: litter number 1 (L#1): 150 days (d), L#2: 39d, L#3: 256d, L#4: 203d, L#5: 43d, L#6: 77d, L#7: 78d, L#8: 63d, L#9: 77d. After weaning age (21d), NAC-treated mice received 1 mg ml^−1^ NAC (A7250, Sigma-Aldrich, St. Louis, MO, USA) dissolved in tap water as their only drinking source for 6–8 weeks, whereas control mice drank tap water. Drinking water was refreshed twice a week, and mice drank on average 10 ml liquid per day (no difference was observed between groups). Overall, 22 male CD1 mice—9 wild type and 13 transgenic, of which 4 were treated with NAC—were anesthetized with inhalation of isoflurane, followed by intraperitoneal injection of an anesthetic mixture (containing 8.3 mg ml^−1^ ketamine, 1.7 mg ml^−1^ xylazin-hydrochloride, 0.8 mg ml^−1^ promethazinium-chloride). Animals were perfused transcardially with 0.1 M phosphate buffer (PB, pH 7.4), followed by fixative for 40 min and finally PB for 10 min. Fixative contained 4% paraformaldehyde or 2% glutaraldehyde and 2% paraformaldehyde in PB (for synapse morphology and synapse stereology experiments). Coronal hippocampal sections were cut on Vibratome (VT1200S, Leica Biosystems, Nussloch, Germany). Fifty-micrometer-thick sections were washed in PB, cryoprotected in 30% sucrose solution, put in vials, frozen in liquid nitrogen and stored at −80 °C until further use. Two hundred fifty-micrometer-thick sections were processed immediately for freeze-substitution and low-temperature Lowicryl embedding directly after perfusions.

### Synapse morphology measurements

A series of 50-μm-thick sections—250 μm apart from each other—were collected from the entire rostrocaudal extent of the hippocampi (from Bregma −0.95 mm, to −4.03 mm) of 19 animals. After washing in PB, sections were treated with 0.5% osmium-tetroxide for 20 min, were dehydrated and embedded in Durcupan. During dehydration, sections were treated with 1% uranyl acetate. After polymerization, we prepared 70-nm-thick sections (Leica EM UC6) from the outer two-thirds of the molecular layer of DG, picked them up on formvar-coated single-slot copper grids and examined them using a Hitachi H-7100 (Hitachi, Tokyo, Japan) electron microscope (EM) and a Soft Imaging System Veleta CCD camera (EMSIS, Münster, Germany). Synaptic area was measured from serial sections of three-dimensional (3D) reconstructed synapses. We only included synapses that were cut near perpendicularly to the sectioning plane. After checking that the series of sections contained the whole synapse, the synaptic area was calculated by measuring the length of the synaptic active zone on each individual section of the same synapse, and then the sum was multiplied by the thickness of the sections (70 nm). The extent of the synaptic active zone was determined based on the extent of the postsynaptic density (PSD). For measurements, we used ImageJ (Fiji). For synaptic cleft width and PSD thickness measurements, synaptic and postsynaptic grayscale values were investigated. The distance between the two highest local grayscale maxima represented the actual synaptic cleft width, and the distance between the highest postsynaptic local maximum and the first local minimum represented the PSD thickness.

### Post-embedding immunoelectron microscopy

Two hundred fifty-micrometer-thick sections from 11 animals were cryoprotected in 10 and 30% sucrose solutions. After slam-freezing on liquid nitrogen-cooled copper blocks, low-temperature dehydration was started at −90 °C with dried methanol containing 0.5% uranyl acetate for 24 h. Freeze substitution was performed at −45 °C using ascending mixtures of methanol and Lowicryl HM20 resin, and finally pure Lowicryl. Then, resin was polymerized using ultraviolet light. Seventy-nanometer-thick sections were picked up on formvar-coated single-slot nickel grids, blocked for 1 h and incubated on drops of primary antibodies overnight. The blocking solution, which was also used for diluting the primary and secondary antibodies, contained 2% human serum albumin and 0.03% Triton X-100 in Tris buffered saline. After incubation in primary antibody (rabbit anti-GluN1, 1:30, gift of professor Masahiko Watanabe) for 18 h, sections were washed in Tris buffered saline and incubated for 5 h on drops of secondary antibody (10-nm gold particle-conjugated goat anti-rabbit, British Biocell International, Cardiff, UK) diluted in blocking solution (1:100), which contained polyethylene glycol (0.5 mg ml^−1^). After washing in ultrapure water, sections were contrasted with saturated aqueous uranyl acetate. The specificity of the primary antibody has been tested by the laboratory of origin.^39^ The omission of the primary antibody resulted in no labeling. Forty-nanometer-wide bands were chosen on the two sides of the postsynaptic membrane as an area representing postsynaptic membrane-associated gold particle labeling. Synaptic areas were measured and synaptic gold particles were counted on each 3D-reconstructed synapse.

### Stereology

The total volume of the outer two-third of the DG molecular layer was determined using the Stereo Investigator system (MBF Bioscience, Williston, VT, USA) in nine animals. From this volume, small regions were chosen randomly and 70-nm-thick section series were examined in EM using the physical disector method. Asymmetric synapses were collected within the counting frame (4 × 4–5 × 5 μm). From the physical disector volume and number of synapses, we calculated synapse density, and multiplied it by the volume of the outer two-third of the DG molecular layer to get the total synapse number.

For the stereological measurement of the number of granule cells, another systematic random series of 50-μm-thick sections—250 μm apart from each other—were collected from the entire hippocampi of 12 animals. Sections were washed in PB, treated with 0.3% saponin in PB, incubated in Mayer's Hematoxylin solution (DAKO, Glostrup, Denmark; diluted 1:20 in PB) for 30 min, washed in running tap water for 10 min, rinsed in distilled water, washed in PB and mounted and coverslipped with Aqua-Poly/Mount (Polysciences, Hirschberg an der Bergstrasse, Germany). Stereology was performed on a Zeiss Axioskop 2 mot plus microscope, using a Plan-Apochromat × 100/1.4 oil immersion objective, Q-Imaging CCD camera and Stereo Investigator software (MBF Bioscience). The optical fractionator method was used as described earlier.^40^ Section thickness was checked in every counting frame (7 × 7 μm, height: 25 μm); *x–y* step size was 100 μm. At least 7-μm-thick volume at the top and bottom of every section was kept as guard zones.

### Analysis

WT and G72Tg animals were selected randomly, after we identified litters, in which sufficient numbers of both WT and G72Tg mice were present. Sample sizes were estimated based on pilot investigations. In all five experiments, described in the five Results sections, investigators were blinded to the group allocations during the experiment and while assessing the outcomes. Data populations with Gaussian distributions (tested with Shapiro–Wilks *W-*test) were compared with parametric tests and other distributions were compared with non-parametric tests. The Mann–Whitney *U*-test was used for the analysis of synaptic area, synaptic cleft width and PSD thickness. Spearman R correlation was used for the analysis of the synaptic GluN1 content. Two sample *t*-test was used to compare total synapse number of different groups, and Friedman test was used for the analysis of granule cell numbers. The null hypothesis was rejected when the *p-*level was under 0.05, and, in such cases, the differences were considered significant throughout this paper.

## Results

### G72Tg animals have larger glutamatergic synapses in DG and it is restored by NAC treatment

**Figure 1:**
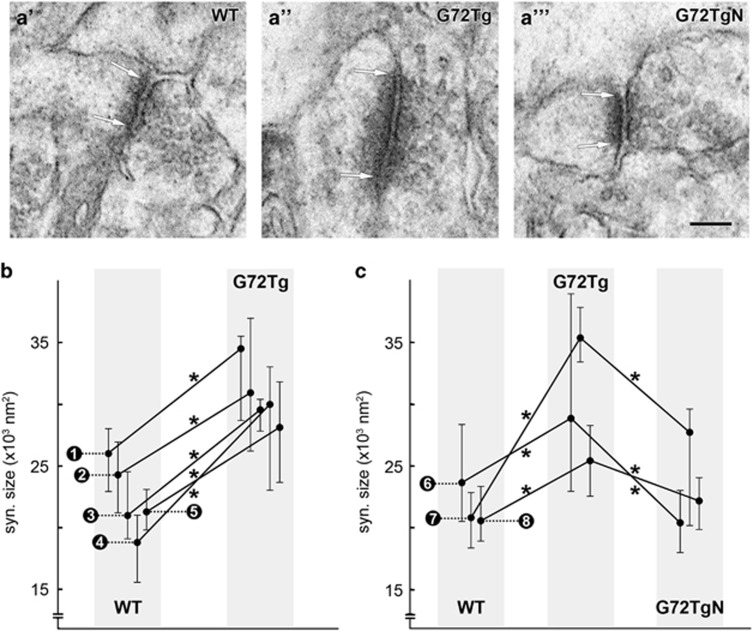
G72Tg mice have larger synapses, the sizes of which are normalized by *N*-acetylcysteine (NAC) treatment. (**a**) Electron micrographs show representative glutamatergic excitatory synapses sampled from the outer two-thirds of the dentate gyrus molecular layer from wild-type (WT, **a'**), G72 transgenic (G72Tg, **a''**) and NAC-treated G72 transgenic (G72TgN, **a'''**) animals (synaptic active zone edges are indicated with arrows). Scale bar: 200 nm for all images. (**b**,**c**) Dots represent the median values of 3D-reconstructed synaptic areas in individual animals, and whiskers mark 40–60 percentile boundaries. Effects were tested in five littermate pairs (**b**, their values are connected) and in three littermate pairs (**c**), and then the *G72* effect was reversed by the NAC treatment in G72TgN littermates (**c**; *P<0.05). Numbers indicate litters. Litter number 1 (L#1): WT: *n*=32 3D-reconstructed synapses, G72Tg: *n*=24, *P*=0.013; L#2: (*n*=52, 38), *P*=0.024; L#3: (*n*=43, 28), *P*=0.014; L#4: (*n*=24, 14), *P*=0.029; L#5: (*n*=30, 59), *P*=0.003; L#6: WT (*n*=62), G72Tg (*n*=48), G72TgN (*n*=87), WT versus G72Tg *P*=0.042, G72Tg versus G72TgN *P<*0.001; L#7: (*n*=69, 49, 51), WT versus G72Tg *P<*0.001, G72Tg versus G72TgN *P*=0.004; L#8: (*n*=60, 56, 78), WT versus G72Tg *P*=0.046, G72Tg versus G72TgN *P*=0.031.

The synaptic active zone area of randomly sampled synapses was measured in the outer two-thirds of the DG molecular layer because this area is the termination zone of the perforant path, and 85% of all asymmetric synapses here originate from these fibers.^41^ Synapses were 3D-reconstructed from serial electron microscopic sections (about two to five section per synapse; Figure 1a), and synaptic areas were calculated accordingly (see Materials and Methods).

First, we measured synaptic areas of five WT and G72Tg littermate pairs, and found a significant increase of synaptic area in G72Tg animals in all five investigated littermate pairs (L#1: WT: *n*=32 3D-reconstructed synapses, median=25.97 × 10^3^ nm^2^, G72Tg: *n*=24 synapses, median=34.46 × 10^3^ nm^2^, *P*=0.013; L#2: WT (*n*=52, 24.25 × 10^3^ nm^2^), G72Tg (*n*=38, 30.89 × 10^3^ nm^2^), *P*=0.024; L#3: WT (*n*=43, 20.96 × 10^3^ nm^2^), G72Tg (*n*=28, 29.51 × 10^3^ nm^2^), *P*=0.014; L#4: WT (*n*=24, 18.77 × 10^3^ nm^2^), G72Tg (*n*=14, 29.95 × 10^3^ nm^2^), *P*=0.029; L#5: WT (*n*=30, 21.26 × 10^3^ nm^2^), G72Tg (*n*=59, 28.09 × 10^3^ nm^2^), *P*=0.003). In the pairwise comparisons of littermates, the median increase was 33%, min–max: 27–60% (Figure 1b). The ages of the littermates (see Materials and Methods) did not correlate either with synaptic area or with the extent of the observed changes (data not shown).

In the second set of experiments, we examined the effect of NAC treatment on changes in synaptic areas (Figure 1c). We used three groups of littermates, each consisting of a WT, a G72Tg and a NAC-treated G72Tg (G72TgN) animal. We found again that synaptic areas were significantly increased in G72Tg animals, and values in each of these littermate groups significantly decreased back to the normal level in NAC-treated G72TgN mice (L#6: WT (*n*=62, 23.61 × 10^3^ nm^2^), G72Tg (*n*=48, 28.83 × 10^3^ nm^2^), G72TgN (*n*=87, 20.36 × 10^3^ nm^2^), WT versus G72Tg *P*=0.042, G72Tg versus G72TgN *P<*0.001; L#7: WT (*n*=69, 20.79 × 10^3^ nm^2^), G72Tg (*n*=49, 35.33 × 10^3^ nm^2^), G72TgN (*n*=51, 27.68 × 10^3^ nm^2^), WT versus G72Tg *P<*0.001, G72Tg versus G72TgN *P*=0.004; L#8: WT (*n*=60, 20.53 × 10^3^ nm^2^), G72Tg (*n*=56, 25.40 × 10^3^ nm^2^), G72TgN (*n*=78, 22.14 × 10^3^ nm^2^), WT versus G72Tg *P*=0.046, G72Tg versus G72TgN *P*=0.031; Figure 1c).

Because synaptic areas of different mice in the same groups (WT, G72Tg or G72TgN) did not differ significantly, we pooled synaptic areas within these groups. The median synaptic area was 22.15 (interquartile range: 16.48–30.90) × 10^3^ nm^2^ (*n*=386) in WT mice, 29.74 (21.74–43.00) × 10^3 ^nm^2^ (*n*=316) in G72Tg mice and 21.89 (16.75–28.98) × 10^3 ^nm^2^ (*n*=216) in G72TgN mice. This means that the synaptic area increased by 33% in G72Tg animals, compared with WT mice (*P<*0.001), and decreased by 35% (of WT values) in NAC-treated G72TgN mice compared with G72Tg animals (*P<*0.001). These results show that the *G72* gene strongly affects synapses, and the NAC treatment can completely reverse these effects.

### Larger synapses contain more NMDA receptors in G72Tg animals as well

**Figure 2:**
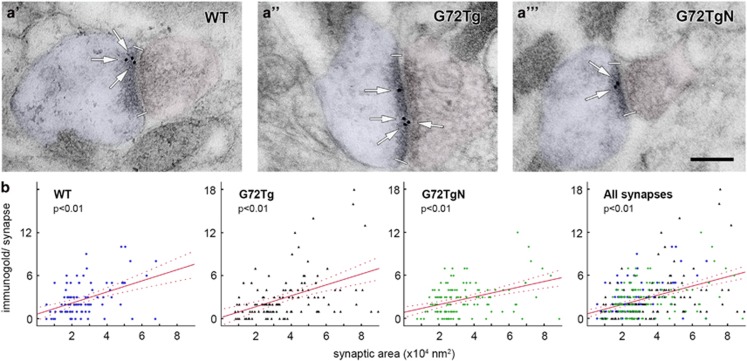
Correlation between synaptic *N*-methyl-d-aspartate (NMDA) receptor content and synaptic area is unaffected by genotype and *N*-acetylcysteine (NAC) treatment. (**a**) Electron micrographs show representative glutamatergic synapses sampled from the outer two-thirds of the dentate gyrus molecular layer from wild-type (WT, **a'**), G72 transgenic (G72Tg, **a''**) and NAC-treated G72 transgenic (G72TgN, **a'''**) animals (synaptic active zone edges are indicated with white bars). Post-embedding immunogold labeling against GluN1 NMDA receptor subunit (arrows). Scale bar: 150 nm for all images. (**b**) Scatterplots show significant (*P<*0.01) correlations between synaptic immunogold particle number and synaptic active zone area in WT (Spearman *R*=0.404, *n*=94 3D-reconstructed synapses), G72Tg (*R*=0.438, *n*=118), G72TgN (*R*=0.346, *n*=129) mice, as well as when data were pooled (*R*=0.385, *n*=341). Animals used for these measurements were taken from litters L#3, L#4, L#6, L#7 and L#8.

NMDARs have heterotetrameric structure with two obligatory GluN1 subunits.^39, 42^ Because NMDAR content correlates with synaptic area, larger synapses contain more NMDARs.^43, 44^ To confirm that such a correlation exists here as well, we carried out quantitative post-embedding immunogold experiments, using a GluN1 subunit-specific antibody. Synaptic area increase in G72Tg animals and the effect of the NAC treatment on synaptic areas could also be observed in these experiments (Figure 2a). We found that synaptic areas and their NMDAR content showed significant correlations (Figure 2b) in WT (*P<*0.001, Spearman *R*=0.404, *n*=94 3D-reconstructed synapses), G72Tg (*P<*0.001, *R*=0.438, *n*=118) and G72TgN animals (*P<*0.001, *R*=0.346, *n*=129) and also when these data were pooled (*P<*0.001, *R*=0.385). According to the latter correlation, 33% increase in synaptic area (described above) corresponds to an ~22% increase in synaptic NMDAR content in G72Tg mice.

### Synaptic cleft width and PSD thickness are unchanged in G72Tg animals

**Figure 3:**
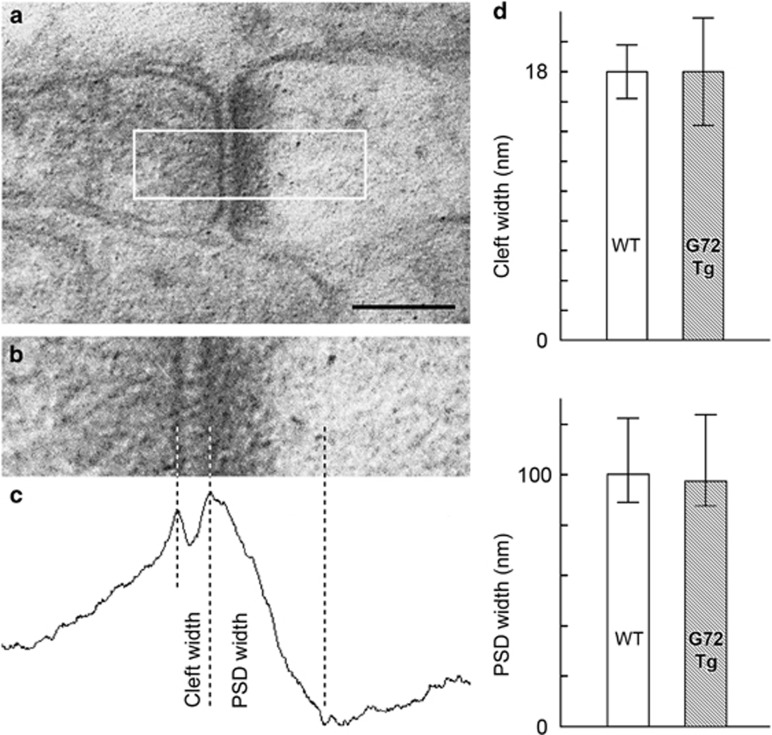
Synaptic cleft width and postsynaptic density (PSD) thickness are unchanged in *G72* transgenic animals. (**a**) Electron micrograph of a glutamatergic synapse sampled for measurement. Scale bar: 150 nm. (**b**) Area selected for measurements (white box in **a**). (**c**) Line graph shows vertically summed optical density values of the synapse shown in **b**. (**d**) Synaptic cleft width (*P*=0.996) and PSD thickness (*P*=0.711) did not differ between wild type (WT; *n*=44 synapses from two mice) and G72Tg (*n*=36 synapses from two mice) mice. The median values and interquartile ranges are shown. Animals used for these measurements were taken from litters L#3 and L#4.

Certain types of synaptic plasticity can influence synaptic scaffolding and synaptic adhesion molecules and can, therefore, influence cleft width and the thickness of PSD. We compared these features between WT and G72Tg animals. Asymmetric synapses were sampled from the outer two-thirds of the DG molecular layer, and examined in the electron microscopic images (Figures 3a and b). The width of the synaptic cleft and the thickness of PSDs were determined by an unbiased algorithm, using the grayscale levels (Figure 3c). We found that neither cleft width (*P*=0.996) nor PSD thickness (*P*=0.711) have changed in the G72Tg mice (Figure 3d). The median synaptic cleft width was 18.0 nm (interquartile range: 16.2–19.8) and PSD thickness was 100.08 nm (88.96–122.32) in WT mice (*n*=44 synapses from two mice). The median synaptic cleft was 18.0 nm (14.4–21.6) and PSD thickness was 97.30 nm (87.57–123.71) in the G72Tg mice (*n*=36 synapses from two mice).

### G72Tg animals have fewer synapses in DG and it is restored by NAC treatment

**Figure 4:**
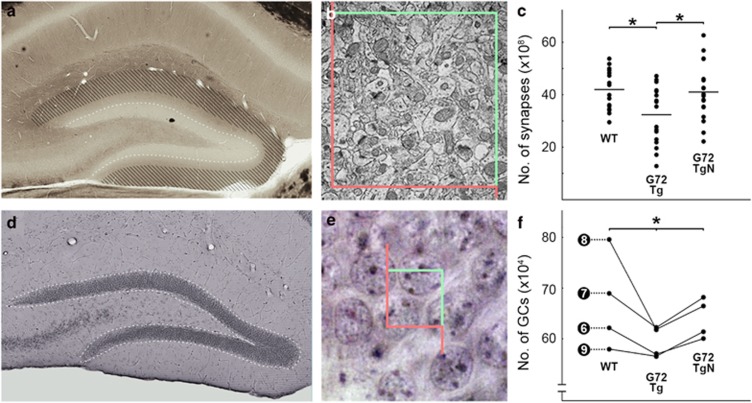
The number of synapses and granule cells decreased in G72Tg mice, and it was normalized by *N*-acetylcysteine (NAC) treatment. (**a**) Light micrograph shows a hippocampal section before measurements. White dashed line marks the molecular and granule cell layer border of the dentate gyrus, stripes indicate the outer two-thirds of the molecular layer, from where the samples were collected. (**b**) Electron micrograph shows an image from a series used for physical disector method. Green/red lines show the stereological counting frame (5 × 5 μm). (**c**) The number of synapses in the outer two-thirds of the molecular layer is decreased in G72Tg mice. This effect is reversed by NAC treatment in G72TgN mice. Dots represent individual measurements (*n*=18 in each group), lines represent the mean values (WT: 41.98 × 10^8^, s.d.=±7.77 × 10^8^; G72Tg: 32.38 × 10^8^, s.d.=±1.07 × 10^9^; G72TgN: 41.04 × 10^8^, s.d.=±1.08 × 10^9^). WT versus G72Tg *P*=0.004, G72Tg versus G72TgN *P*=0.021. Total number of synapses counted during stereological measurements: WT: 444 synapses, G72Tg: 327 synapses, G72TgN: 417 synapses. (**d**) Light micrograph shows the dentate gyrus. White dashed line marks the granule cell layer (the sampling area). (**e**) Light micrograph from optical fractionator measurement. Green/red lines: stereological counting frame (7 × 7 μm). Hematoxylin-labeled nuclei of granule cells (purple) were counted. (**f**) Number of granule cells is decreased in G72Tg mice, which is reversed by NAC treatment (*, Friedman test *P<*0.05). Note that the optical fractionator method used in this experiment results in a single final value for every individual animal investigated (dots). Values connected by lines correspond to littermates. Numbers indicate litters. Litter number 6 (L#6): WT: number of cells counted during measurement: *n*=380, G72Tg (*n*=349), G72TgN (*n*=374); L#7: *n*=369, 335, 370; L#8 *n*=392, 344, 352; L#9: *n*=297, 324, 348. Data are given for one brain hemisphere in all cases.

We tested whether the expression of the *G72* gene affects the number of synapses. Using quantitative stereological measurements with physical disector method, we estimated the number of synapses in the perforant path recipient outer two-thirds of the DG molecular layer (Figures 4a and b) in three triplets of littermates (L#6–8), by taking 18 volume samples per groups (WT, G72Tg, G72TgN). Compared with WT mice (mean= 41.98 × 10^8^, s.d.=±7.77 × 10^8^, a total of *n*=444 synapses counted), synapse number decreased in G72Tg mice (mean=32.38 × 10^8^, s.d.=±1.07 × 10^9^, *n*=327, WT versus G72Tg *P*=0.004) and restored near to the normal level after NAC treatment (mean=41.04 × 10^8^, s.d.=±1.08 × 10^9^, *n*=417, G72Tg versus G72TgN *P*=0.021, Figure 4c).

### G72Tg animals have fewer granule cells in DG and it is restored by NAC treatment

Using quantitative stereological measurements with optical fractionator method (Figures 4d and e), we tested whether the expression of the *G72* gene affects the number of granule cells in DG. Estimations are given for one brain hemisphere in four groups of littermates. Compared with WT mice, the number of cells decreased in all G72Tg mice (with a median of 9%) and restored near to the normal level after NAC treatment (with a median of 7%). The values were 62.13 (WT, *n*=380 sample of cells), 56.96 (G72Tg, *n*=349) and 60.03 (G72TgN, *n*=374) × 10^4^ cells in the first group (L#6); 68.98 (WT, *n*=369), 62.22 (G72Tg, *n*=335) and 68.13 (G72TgN, *n*=370) × 10^4^ cells in the second group (L#7); 79.55 (WT, *n*=392), 61.87 (G72Tg, *n*=344) and 66.44 (G72TgN, *n*=352) × 10^4^ cells in the third group (L#8); and 57.98 (WT, *n*=297), 56.56 (G72Tg, *n*=324) and 61.39 (G72TgN, *n*=348) × 10^4^ cells in the fourth group (L#9) of littermates (Figure 4f). These changes among littermates are statistically significant (*P*=0.039).

## Discussion

Here we demonstrated that *G72* gene expression in a humanized schizophrenia model mouse induces synaptic and cellular changes, and found that these changes are normalized by NAC treatment. Our major findings are that (1) in G72Tg mice, glutamatergic synapses are significantly larger in the main input zone of DG compared with WT littermates and (2) these larger synapses contain more NMDA receptors, whereas receptor density remains unchanged. On the other hand, (3) the number of excitatory synapses targeting DG granule cell spines is decreased in G72Tg animals and (4) there are fewer granule cells in DG. (5) Most importantly, treatment with NAC was efficient in normalizing all these alterations, suggesting that oxidative stress has a major role in *G72* gene-induced pathology.

*G72* gene polymorphisms confer susceptibility to schizophrenia^13^ and bipolar disorder.^29, 30, 45^ Schizophrenic patients with *G72* gene polymorphisms have an elevated level of LG72 protein in the brain and plasma.^15, 16^ G72Tg mice express *G72* transcripts similar to those in humans, including the longest open reading frame, LG72.^22^ Expression of *G72* transcripts was highest in DG,^22^ a brain area frequently affected in schizophrenia and bipolar disorder (see Introduction). G72Tg mice show schizophrenia-like behavioral alterations, including a massive DG-dependent deficit in spatial learning ability.^23^ These hippocampal circuits, responsible for learning and memory, are mainly glutamatergic. The ‘glutamate hypothesis' of schizophrenia suggests a dysregulation of the glutamatergic system with a hypofunction of NMDA-type ionotropic glutamate receptors, along with changes in the number of NMDARs in specific brain regions.^4, 5^ In schizophrenia, many glutamate-regulated processes seem to be perturbed, which lead to a decreased number of excitatory synapses resulting in insufficient neuronal circuits.^2^ This is exactly what we found in G72Tg mice. Similar changes may occur in patients with *G72* gene polymorphisms with consequently higher LG72 protein levels.

### Unlikely effect of *G72* on DAO in DG

Previously, LG72 was considered to be a modulator of the D-serine-degrading enzyme, DAO.^14, 18, 19^ D-serine and glycine are NMDAR co-agonists that have a common binding site on NMDARs. In the hippocampal formation, D-serine seems to be the main co-agonist of NMDARs because its density is high in the hippocampus and the outer two-thirds of the DG molecular layer, whereas glycine is absent in these regions.^46, 47^ D-serine is essential for the physiological function of the adult DG and it is also necessary for full NMDAR function.^48^ A reduction in D-serine levels can be observed in patients with schizophrenia^49, 50^ and treatment with this amino acid results in ameliorated symptomatology.^51, 52^ However, only low levels of DAO are present in the molecular layer of DG,^53^ and the decrease in the D-serine level observed in aging or under pathological conditions is probably because of an impairment in serine racemase activity^54^ rather than because of DAO. Therefore, it is unlikely that the changes observed in this study are consequences of G72-triggered altered DAO activity.

### Possible role of *G72* in the regulation of oxidative stress

LG72 has been shown to be located in mitochondria.^21^ Recently, the mitochondrial enzyme methionine-R-sulfoxide reductase B2 has been identified as a binding partner of LG72, suggesting that LG72 can influence oxidative stress regulation.^20^ Subcellular localization and the ability of LG72 to bind flavin-containing proteins also suggest a connection between LG72 and complex I of the mitochondrial respiratory chain.^23^ Complex I is often affected in schizophrenia and bipolar disorder.^55, 56^ Impaired complex I function results in increased generation of reactive oxygen species.^32^ Because complex I is highly susceptible to oxidative damage,^57^ this leads to a vicious circle and results in the depletion of antioxidants. Consequent oxidative stress has a role in the generation of both schizophrenia and bipolar disorder.^58, 59, 60^ This is represented as a reduction in the GSH level in patients suffering from these diseases.^33, 34^ G72 transgenic mice show signs of altered mitochondrial function and oxidative stress.^23, 61^ Furthermore, these mice also possess decreased complex I activity and a reduction in the GSH level.^23^ The effectiveness of the putative GSH precursor NAC in ameliorating cognitive,^23^ cellular and subcellular alterations of G72Tg mice (present study) confirms the possible role of *G72* transcripts in the regulation of oxidative stress.

NMDARs have a redox-sensitive modulatory site. Oxidative environment can inhibit NMDAR function up to 50%.^62^ Depleted GSH level does also have a direct effect on NMDARs,^63^ whereas the GSH level is decreased in G72Tg mice, similar to patients suffering from schizophrenia or bipolar disorder. Although we did not assess NMDAR hypofunction here directly, the potential link between NMDAR hypofunction and oxidative stress in the pathogenesis of schizophrenia has also been proposed before.^64^ Here, we hypothesize that larger synapses with more NMDARs observed in the molecular layer of G72Tg mice are a response to NMDAR hypofunction due to oxidative damage. These larger synapses are necessary and sufficient to maintain basal synaptic activity; however, they are unable to adapt to further potentiation demands, resulting in decreased plasticity and cognitive deficits of these mice. In addition, although changes in the synaptic structural profile often accompany physiological alterations of neurons,^65, 66, 67^ we found no difference in synaptic cleft width or PSD thickness of G72Tg mice compared with WT animals, further suggesting an intact synaptic architecture, but changed NMDAR-related functions.

Furthermore, damaged presynaptic mitochondrial activity may also have a role in the observed changes. Electrophysiological investigations of these synapses revealed unchanged basal activity, but a deficit in sustaining high-frequency transmission and in paired-pulse facilitation,^23^ suggesting a presynaptic impairment and damaged mitochondrial function. This may also result in a postsynaptic compensatory change that helps to maintain a certain level of synaptic activity by increasing synaptic size and receptor content.

We found a decrease in the number of granule cells (~9% decrease) and an even larger decrease in their synapses (~23% decrease), which suggests that the decline in the number of granule cells does not account for all the losses of excitatory synapses on their dendrites. This means that, on average, each individual granule cell receives less glutamatergic input in G72Tg animals compared with WT mice. The observed changes may be either because of an NMDAR hypofunction or are directly induced by oxidative stress in the DG. Granule cells are continuously generated in DG, even in adulthood, and newborn cells are indispensable for circuit formation and essential for intact learning and memory processes.^68^ The survival of newborn neurons depends on proper NMDAR function.^69^ On the other hand, neurogenesis is highly sensitive to oxidative stress.^70, 71^ Furthermore, oxidative environment promotes the aging of cells, shortening their lifetime.^71^ Decreased neurogenesis has been linked to schizophrenia,^72^ whereas neuronal atrophy is thought to accompany bipolar disorder.^73^ These processes can lead to a lower number of dentate granule cells, which we found in G72Tg animals, whereas insufficient synaptic connectivity in the DG may be responsible for impaired spatial learning ability of these mice.

### Beneficial effects of NAC treatment

NAC is currently used in acetaminophen overdose and as a mucolytic agent. It provides protection against oxidative stress, can enhance cystine-glutamate antiporter, reduce inflammatory cytokines and has been trialed in several psychiatric disorders.^74^ NAC was effective in bipolar disorder,^37, 38^ and also in ameliorating negative symptoms in patients with schizophrenia.^35, 36^ In G72Tg animals, the reduced GSH level could be rescued by treatment with NAC.^23^

In the present study, we have shown that oral NAC treatment can effectively normalize cellular and synaptic alterations of G72Tg mice, and we present the first high-resolution anatomical evidence that NAC can directly act at cellular and synaptic levels. If the presence of G72-susceptibility polymorphisms could be routinely detected by responsible genetic profiling in schizophrenia or bipolar disorder patients, NAC treatment could provide a personalized causal treatment for deficits caused by *G72* polymorphisms, and these human patients may see an improvement in negative and cognitive deficits—a field where most current antipsychotics fail. These beneficial effects could be achieved with the repurposing of a cheap, simple and safe drug, which is already used in other fields of medicine.
